# Moving the furniture around – how herpes simplex virus 1 remodels the host nucleus

**DOI:** 10.1080/19491034.2026.2761149

**Published:** 2026-11-11

**Authors:** Shani Nadav-Eliyahu, Yaron Shav-Tal

**Affiliations:** The Mina & Everard Goodman Faculty of Life Sciences & Institute of Nanotechnology, Bar-Ilan University, Ramat Gan, Israel

**Keywords:** HSV1 infection, nuclear organization, nuclear speckles, RNA export, nuclear bodies

## Abstract

Herpes simplex virus 1 (HSV-1) is a nuclear-replicating DNA virus that remodels the host cell nucleus to support viral gene expression, genome replication, and production of infectious progeny. During infection, HSV-1 alters chromatin organization, nuclear biophysical properties, and nuclear bodies composition and dynamics. These changes reflect viral strategies to counteract intrinsic anti-viral defenses and reorgenaize host nuclear functions to favor infection. Viral replication compartments reshape the nuclear environment and interact with nuclear bodies involved in RNA metabolism and gene regulation. HSV-1 mRNAs, many of which are intronless, rely on host export pathways adapted by viral factors such as ICP27, which links viral transcripts to host export receptors including NXF1. Recent evidence suggests that nuclear speckles may serve as specialized intranuclearhubs for immediate-early viral transcripts before export. This review discusses how HSV-1 reorganizes nuclear architecture and nuclear bodies, to regulate viral gene expression, RNA processing and nuclear export.

## Introduction

The genetic material in the cell nucleus is organized in chromosomes that have complex 3D organization and are organized into distinct regions in the nucleus known as chromosome territories (CTs) [1]. Chromosome conformation capture approaches such as Hi-C have unveiled chromatin organization into A and B compartments, where the A compartment corresponds to active chromatin regions and the B compartment exhibits repressive characteristics [2]. The nucleoplasm, or interchromatin space contains different structures which are physically and functionally distinct, known as nuclear bodies (NBs) [3]. These usually contain distinct sets of proteins and RNAs, specific to each type of nuclear body. For example, ribosomal RNA (rRNA) and Nop1p/fibrillarin are attributed to nucleoli, while MALAT1 long non-coding RNA (lncRNA) and SR proteins are attributed to nuclear speckles. Because nuclear bodies are membrane-less, they are considered ‘liquid-liquid phase separated condensates’ or ‘nuclear biomolecular condensates’ [4]. Many studies now demonstrate the strong and intricate connection between gene loci, nuclear bodies and biophysical properties of the nucleus, essentially affecting genome architecture and function [5–14].

Nuclear bodies are dynamic structures often composed of multiple layers, each defined with unique components [15]. Moreover, different proteins and RNAs can exit the NB to the surrounding nucleoplasm, or to another NB, allowing the adaptation of NBs in morphology and function to different cellular contexts [16]. It is known that nuclear architecture and subsequently NBs can be altered in response to different cellular stimuli such as heat shock, oxidative stress and viral infection [17].

This review focuses on host nuclear remodeling during lytic HSV-1 infection in epithelial cells and fibroblasts. The conclusions discussed are therefore primarily applicable to these experimental systems and should not be assumed to extend to neuronal infection or latency.

## HSV-1 affects nuclear architecture

*Herpes simplex* Virus type 1 (HSV-1), which belongs to the *Herpesvirales* order, is a double-stranded DNA virus that must enter the nucleus to express its genes. Importantly, upon infection, HSV-1 dramatically alters nuclear organization and structure. After entry into the cytoplasm, the herpesvirus capsid is transported along microtubules to the nucleus. Upon docking at the nuclear pore complex, the capsid undergoes uncoating, and the viral DNA is released through the nuclear pore into the nucleus [18,19]. Electron microscopy has shown that capsids dock at the nuclear pore, after which the viral DNA translocates through the pore in a highly compact, rod-like form, where the empty capsid is released [20–23]. Once inside the nucleus, the viral genome adopts a circular form for replication and gene expression. Viral gene expression occurs in a highly regulated and sequential manner, in which the expression of one set of genes leads to the expression of the next set. The viral protein VP16, a tegument protein, interacts with several cellular proteins to initiate the expression of viral genes [24,25]. Immediate-Early (IE) genes are expressed first, leading to the production of α proteins. These proteins enable the expression of Early (E) genes, which are translated into β proteins. Subsequently, DNA replication begins, and late genes are expressed, leading to the production of γ proteins [26]. Many α and β proteins are enzymes or DNA-binding proteins, whereas most γ proteins are structural proteins.

Viral genes are transcribed by cellular RNA polymerase, with the participation of viral proteins. Herpesviruses differ from other DNA viruses in that they encode numerous enzymes involved in DNA synthesis, and these enzymes serve as good targets for anti-viral drugs. After the synthesis of new viral DNA, it is packaged into capsids in the nucleus. The maturation of the virus occurs by the release of nucleocapsids that exit through the inner nuclear membrane, finally reaching the cytoplasm. Genome-containing HSV-1 nucleocapsids exit the nucleus through a primary envelopment/de-envelopment pathway. Capsids bud through the inner nuclear membrane into the perinuclear space, thereby acquiring a transient primary envelope. This envelope subsequently fuses with the outer nuclear membrane, releasing unenveloped capsids into the cytoplasm [27–29]. HSV-1 remodels the nuclear architecture to allow the capsids to reach the nuclear membrane by diffusion, rather than directed transport [30]. In the cytoplasm unenveloped capsids acquire tegument proteins and subsequently undergo secondary envelopment by budding into trans-Golgi network membranes, yielding infectious particles [31]. Thus, enveloped virus particles move in vesicles toward the cell surface and are released from the cell by fusing with the plasma membrane for further infection of adjacent cells [32].

Infection with HSV-1 leads to dramatic changes in the nucleus, including disassembly of the nucleolus [33] and PML bodies [34], in addition to significant chromatin condensation, resulting in disruption of the nuclear lamina [35–37]. Additionally, crucial cellular processes such as host transcription [38,39], RNA splicing [40], protein synthesis [41], and cellular response to infection are suppressed [42]. The viral genome replication process in infected cells occurs within the nucleus in regions known as viral replication compartments (VRCs). Initially, after the injection of viral DNA into the nucleus, several small VRCs are formed, which subsequently grow and come into close contact, forming larger structures while maintaining distinct viral genome territories [43,44]. The formation and expansion of VRCs are accompanied by significant changes in nuclear architecture, including chromatin compaction and its displacement toward the nuclear periphery [45–48] (Figure 1). Despite this peripheral marginalization, the chromatin layer retains interchromatin channels that allow progeny capsids to access the nuclear envelope [49,50]. These result in alterations in the gene expression profile of the host cell [51]. The virus induces host transcription shut-off for many cellular genes, although some studies have found that the expression of certain genes remains constant or even increases during infection [51–56].

**Figure 1.**
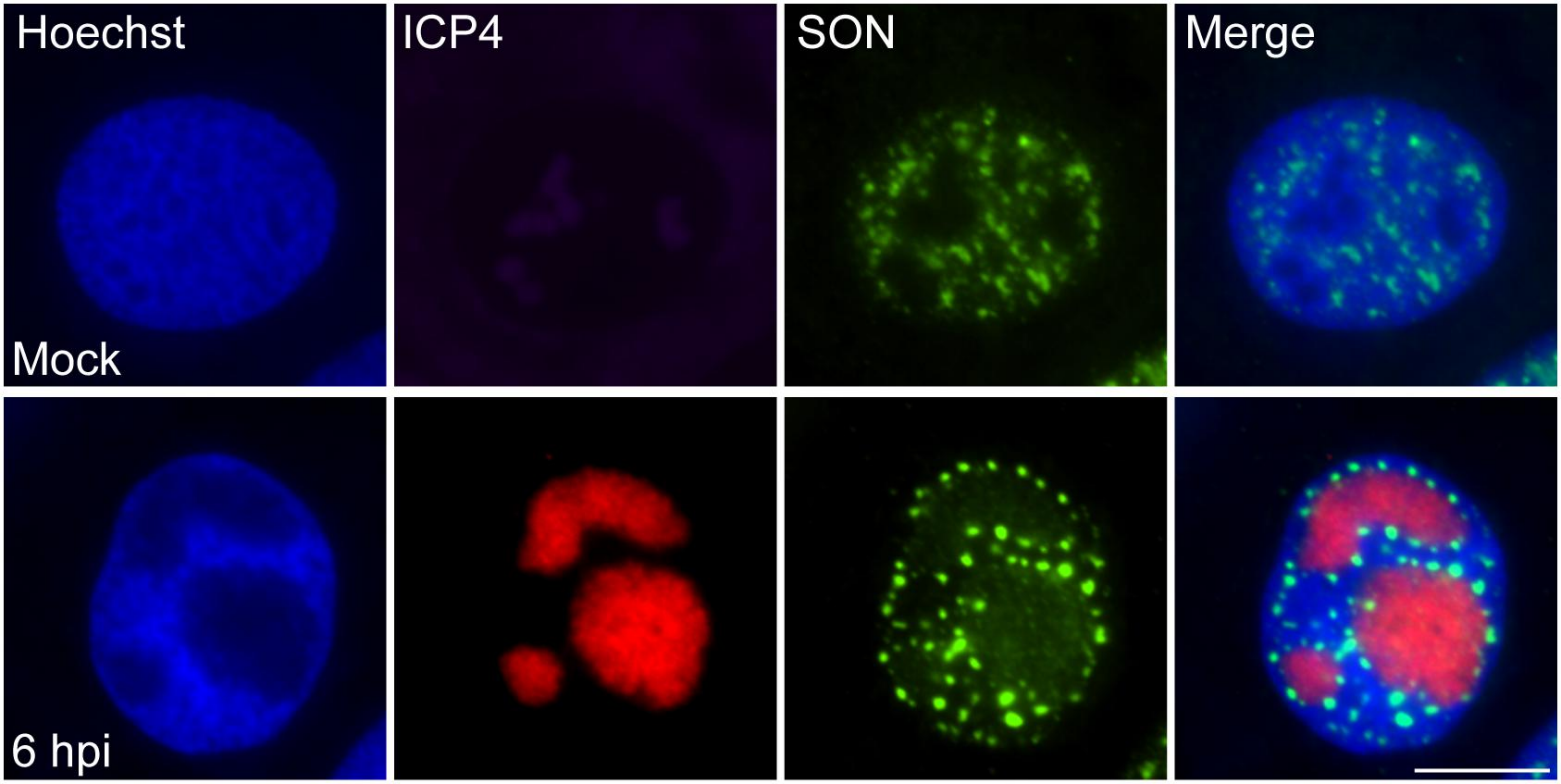
U2OS cell mock infected (top panel) or infected (lower panel) with HSV-1 for 6 hours, stained for the viral protein ICP4 (red), which marks the VRCs, and the cellular protein SON (green), which marks nuclear speckles. The DNA is counterstained with Hoechst (blue). Scale bar = 10 µm.

Other nuclear architecture regulators impaired by HSV-1 infection are CTCF and cohesin, which are central regulators of three-dimensional genome organization. CTCF, together with the cohesin complex, contributes to the formation and stabilization of chromatin loops and topologically associating domains (TADs), thereby bringing otherwise distant genomic regions into spatial proximity and regulating their functional interactions. Within cohesin, RAD21 is a core subunit that contributes to the integrity and chromatin-associated functions of the complex. Importantly, recent work has extended the role of CTCF-cohesin beyond canonical chromatin looping by demonstrating that CTCF and cohesin, including RAD21, contribute to the spatial association of chromatin with nuclear speckles. In particular, a putative speckle-targeting motif within RAD21 was shown to be required for efficient chromatin–nuclear speckle association, suggesting that cohesin can contribute to the spatial organization of chromatin relative to nuclear bodies independently of its canonical role in loop extrusion [57].

HSV-1 infection profoundly remodels this architectural system. During lytic infection, CTCF is recruited to multiple sites across the HSV-1 genome and localizes to developing VRCs, where its depletion reduces viral transcription, viral genome replication, and virus production. These effects have been associated with altered RNA polymerase II occupancy and increased deposition of repressive chromatin marks on the viral genome [58]. Similarly, cohesin components, including SMC1, SMC3, and RAD21, are recruited to HSV-1 replication compartments, where cohesin depletion impairs RNA polymerase II association with viral genes and reduces viral transcription [59]. Notably, CTCF and cohesin recruitment to VRCs appears to be at least partly independent, suggesting that their functions during lytic infection may extend beyond a canonical CTCF-dependent cohesin-loading mechanism [59].

Recent work has further demonstrated that HSV-1 infection extensively remodels the three-dimensional organization of the host genome. Using chromosome-conformation capture and super-resolution imaging, it was shown that HSV-1 infection induces extensive host chromatin condensation accompanied by a progressive loss of RNA polymerase II, TOP1, and cohesin from host chromatin and their redistribution to viral replication compartments (VRCs). Within VRCs, newly replicated viral genomes were found to be in close association with RNA polymerase II and cohesin. Consistent with this redistribution, HSV-1 infection resulted in dynamic rearrangement and at late post-infection time points, substantial loss of host TADs and chromatin loops, whereas A/B compartment identity was comparatively preserved. These findings support a model in which HSV-1-induced sequestration of nuclear transcriptional and chromatin-associated machinery into VRCs contributes to the large-scale reorganization and compaction of host chromatin [38].

Multiple CTCF insulator sites have been identified around the LAT (Latency-associated transcript) and immediate-early regions of the HSV-1 genome, and RAD21 is enriched near several of these sites in latently infected sensory neurons. These observations support a model in which CTCF-cohesin interactions contribute to the higher-order organization of the latent viral genome and may help define distinct chromatin domains encompassing transcriptionally permissive LAT-associated regions and transcriptionally repressed immediate-early loci [60]. Consistent with this model, recent chromosome-conformation analyses identified multiple long-range *cis* interactions between CTCF-associated regions in quiescent HSV-1 genomes, providing direct evidence that CTCF-associated elements participate in the three-dimensional organization of the viral genome [61]. Together, these findings suggest that CTCF and cohesin/RAD21 function not only as regulators of viral transcription but also as components of the higher-order chromatin architecture that shapes the latent HSV-1 genome and its transition toward reactivation.

## Nuclear bodies during HSV-1 viral infection

HSV-1 infection profoundly alters the composition and integrity of various host nuclear bodies. The main nuclear bodies affected by HSV-1 are PML bodies. HSV-1 targets PML bodies that are key mediators of intrinsic antiviral defense that help in sensing and repressing incoming viral genomes, potentially promoting latency-associated silencing [62–65]. Recruitment of the PML repressive proteins (PML, Sp100 and hDaxx) to HSV-1 induced foci is dependent on SUMO interaction [66]. The viral E3 ubiquitin ligase, ICP0, counteracts this restriction by localizing to PML bodies and inducing the degradation of organizer proteins such as PML and Sp100, leading to PML body disassembly, with subsequent recruitment of PML protein to late-stage VRCs [67–69]. Although incoming HSV-1 genomes rapidly associate with PML bodies, this interaction is transient due to ICP0-mediated disruption, which prevents genome entrapment and silencing [70,71]. In the absence of ICP0, this restriction becomes especially evident, as ICP0-null HSV-1 has a high probability of becoming transcriptionally repressed and entering quiescence rather than progressing efficiently through lytic infection.

PML depletion increases the gene expression and plaque-forming efficiency of ICP0-null virus, supporting the idea that PML contributes to a repression mechanism normally counteracted by ICP0 [72]. However, PML bodies have a dual role during HSV-1 infection [71,73]. On one hand, PML, Sp100, Daxx, ATRX, and IFI16 are recruited to incoming viral genomes and can promote chromatinization, genome entrapment, and transcriptional repression, particularly in the absence of ICP0 [74–78]. On the other hand, PML-related functions can also support efficient HSV-1 gene expression and replication-compartment formation. In PML-knockout HEp-2 cells infected with wild-type HSV-1 at a low multiplicity of infection, viral yields were lower than in parental HEp-2 cells, and ICP0 accumulation was delayed [71]. This defect was not observed at high multiplicity, suggesting that PML can facilitate efficient initiation of lytic infection under low-genome-input conditions. Similarly, in primary human foreskin fibroblasts infected with an ICP0-null HSV-1 mutant, siRNA-mediated PML depletion reduced ICP27 and ICP8 expression, impaired viral DNA replication, and decreased the fraction of cells forming defined ICP8-positive replication compartments, indicating that a PML-related function can promote replication-compartment maturation in this context [77]. In contrast to PML, SP100 appears to predominantly restrict HSV-1 replication, as SP100 depletion increased viral replication compartment formation and viral yields at low multiplicities of infection [79]. Thus, PML bodies are not simply antiviral structures, and their effects depend on PML isoform composition, cell type, viral genotype, and the stage of infection [71,73,77].

HSV-1 infection causes dramatic modifications to nucleolar architecture, including disaggregation of the nucleolus and profound alterations in nucleolar morphology. The virus encodes at least 12 proteins that specifically localize to the nucleolus and alter its structure and function by redistributing nucleolar proteins. The three most abundant nucleolar proteins, nucleolin, B23 (nucleophosmin/NPM1), and fibrillarin, are redistributed out of the nucleoli as a consequence of HSV-1 infection [33]. Nucleolin shows progressive increase in expression during infection and relocalizes to viral replication compartments, where it colocalizes with the viral protein ICP8 during active viral DNA replication [33]. Additionally, nucleolin participates in nucleocapsid transport from the nucleus to the cytoplasm and is required for HSV-1 nuclear egress [80]. The viral protein UL24 has a profound effect on nucleolar architecture, with UL24’s N-terminus responsible for the redistribution of nucleolin and B23 [81,82]. Kaposi’s sarcoma-associated herpesvirus (KSHV), a gammaherpesvirus, has also been shown to extensively disrupt nucleolar organization during lytic infection [83] and to affect nuclear speckles [84].

Although HSV-1 extensively remodels nuclear bodies, its direct functional relationship with Cajal bodies is less defined. The HSV-1 protein ICP0 has been reported to induce redistribution of coilin, the Cajal body marker, together with other nuclear-body-associated factors such as SMN (component of gems -Gemini of Cajal bodies) and the nucleolar protein fibrillarin, during an interphase centromere damage response. Thus, HSV-1 may perturb Cajal body-linked proteins indirectly [85,86].

Other nuclear bodies that are altered by HSV-1 are the nuclear speckles and paraspeckles. Nuclear speckles are dynamic, membrane-less nuclear condensates enriched in pre-mRNA splicing factors and other RNA-processing proteins, with SON and SRRM2 proteins serving as core components [87–90]. Besides RNA-processing proteins, nuclear speckles are also known to contain different RNA molecules [91], among them is the long non-coding RNA MALAT1. MALAT1 can modulate the partitioning of nuclear speckle proteins into distinct subdomains, highlighting an RNA-dependent layer of nuclear speckle organization [92]. Paraspeckles are distinct nuclear bodies found near nuclear speckles and are built on the lncRNA NEAT1. They are known to function as stress-responsive gene regulatory bodies by sequestering proteins and RNAs [93,94]. HSV-1 infection was shown to induce the redistribution of nuclear speckles, specifically around viral replication compartments [95–97], as well as the redistribution of paraspeckle proteins and accumulation in VRCs [98,99]. The adjacency of these nuclear bodies to VRCs implies a connection between viral gene loci and viral mRNA transcription, processing and export. SRSF2, a core nuclear speckle protein, was shown to associate with paraspeckle factors including NEAT1, PSPC1, and p54nrb, and this combined network led to histone modifications near HSV-1 promoters, suggesting that HSV-1 uses remodeled nuclear-body interactions to support gene expression [99]. Furthermore, VRCs migrate and merge with nuclear speckle structures to facilitate the export of viral mRNA transcripts [95]. Our recent work shows that HSV-1 reshapes nuclear speckles structurally and functionally, with nuclear speckles increasing in size and number, increased mobility of the SRRM2 core protein, and exclusion of MALAT1 from the condensates during infection. Importantly, viral immediate-early (IE) transcripts were shown to accumulate in nuclear speckles prior to export from the nucleus, and nuclear speckle disruption led to IE viral mRNA retention in the nucleus, impairing viral gene expression progression [97].

Considering the redistribution of nuclear speckles and the alteration of the 3D genome organization regulators CTCF and cohesin, raises an intriguing possibility regarding the relationship between VRC formation and nuclear speckle remodeling. Because CTCF-cohesin/RAD21 contributes to the normal spatial association between chromatin and nuclear speckles, sequestration or redistribution of these architectural factors toward VRCs could potentially perturb the normal coupling between host chromatin and nuclear speckles. In this model, HSV-1-induced recruitment of CTCF and cohesin/RAD21 to VRCs would not only reorganize viral and host chromatin but could also alter the spatial positioning or organization of nuclear speckles within the infected nucleus. Conversely, HSV-1-induced remodeling of the nuclear speckle environment could influence the distribution and activity of chromatin-associated cohesin and CTCF, suggesting a potentially bidirectional relationship between VRC formation, cohesin redistribution, and nuclear-body organization. Although a direct link between cohesin recruitment to VRCs and nuclear speckle remodeling has not yet been established, this model provides a potential mechanistic framework connecting the previously described effects of HSV-1 on CTCF/cohesin, host chromatin architecture, and nuclear speckle organization.

Recent studies show that HSV-1 softens and increases the mesoscale fluidity of the nucleus rather than just changing gene expression [100,101]. Correspondingly, the nuclear elasticity and viscosity during HSV-1 infection are reduced. These findings emphasize that the virus not only hijacks the molecular pathways in the cell but also remodels the mechanical properties of the nucleus to support its life cycle. Specifically, ICP4 is the key viral protein that enables the fluidity of the nucleus, and decreased nuclear fluidity results in reduced viral titer [100,102]. Moreover, ICP4-dependent nuclear fluidization enables the growth of nuclear condensates. More broadly, the material properties of the nuclear interior can influence condensate behavior: chromatin-poor, mechanically permissive regions favor condensate nucleation and growth, whereas stiffer or more uniformly dense chromatin networks can restrict particle mobility, coalescence, and development. A softer, chromatin-poor environment lowers the energetic cost of nucleation and growth, whereas a stiffer or more uniformly dense chromatin network can restrict particle mobility, slow coalescence, and prevent condensates from ripening into larger bodies [12,14,103]. Therefore, HSV-1-induced chromatin marginalization may further reshape the spatial distribution of chromatin-poor regions during later infection, potentially influencing the nuclear environment in which viral replication compartments mature and promote condensate growth. While this is important for the viral condensate- viral replication compartment, we can also infer that this may have a large impact on nuclear bodies as well. Because nuclear bodies are membrane-less condensates, their assembly likely reflects a balance between biochemical affinity and nuclear mechanics. If the nucleus is more fluid, chromatin-poor regions are larger, condensates can sample more space, fuse more readily, and reorganize faster. In our recent study, we observed that the size and dynamics of nuclear speckles increased during HSV-1 infection [97], which may also be explained by increased nuclear fluidity.

In line with the idea that HSV-1 infection promotes remodeling of the nuclear environment and enhances condensate formation, other condensates which appear during infection are VICE domains. VICE (virus-induced chaperone-enriched) domains were first characterized during HSV-1 infection as nuclear foci that sequester molecular chaperones, including Hsc70, Hsp70, Hsp40, and Hsp90, together with ubiquitin-conjugated proteins and proteasome components. Early studies showed that ICP0 is necessary for the early formation of these foci and sufficient to redistribute chaperones when expressed alone, suggesting that VICE domains participate in handling misfolded or damaged proteins during infection [104]. Subsequent work established that VICE domains function as nuclear protein quality control centers and contain misfolded proteins with proteolytic activity, while Hsp90 was linked to the proper nuclear localization of the viral DNA polymerase [105,106]. ICP22 was subsequently shown to be required for VICE-domain formation during productive infection and was sufficient to induce Hsc70-containing nuclear inclusions when expressed alone [107].

More recent work proposes that ICP22 organizes these domains through liquid-liquid phase separation and recruits ubiquitinated RNA Pol II-Ser2P for UL36-mediated deubiquitylation, thereby protecting RNA pol II from degradation and supporting viral transcription [108]. The Sandri-Goldin laboratory further linked ICP27 to VICE domains by showing that ICP27 interacts with Hsc70 and is required for Hsc70 focus formation at the periphery of viral transcription sites [109]. Related work demonstrated that ICP27 interacts with the C-terminal domain of RNA polymerase II, promotes its recruitment to viral transcription sites, and facilitates its proteasomal degradation during infection [110]. Consistent with these functions, ICP27 phosphorylation-site mutants fail to form Hsc70 nuclear foci and show impaired RNA polymerase II colocalization, replication-compartment formation, viral gene expression, and replication [111].

Although VICE domains are not classical constitutive nuclear bodies, they represent HSV-1-induced membrane-less assemblies that accumulate host chaperones and additional nuclear factors during infection. Recent spatial proteomics showed that VICE domains sequester ribosome biogenesis factors away from rRNA, linking HSV-1-induced nuclear condensates to impaired ribosome supply and broader remodeling of host translation [112]. Thus, VICE domains provide an example of how HSV-1 can exploit or induce nuclear condensate-like structures to reorganize host nuclear functions during infection.

In conclusion, HSV-1 infection leads to major changes within the nucleus, affecting nuclear architecture and nuclear bodies. These may reflect functional changes that are required for efficient infection and generation of viral progeny but may also be a manifestation of modulations in molecular interactions and the physical crowding taking place in the nucleus as infection proceeds and viral factories take over the nuclear space.

## Splicing during HSV-1 infection

Another cellular process which is altered during infection is pre-mRNA splicing. The immediate-early protein ICP27 remodels host pre-mRNA splicing through interactions with components of the cellular splicing machinery. Although early studies demonstrated ICP27-dependent splicing inhibition, subsequent transcriptome-wide analyses support a more selective model, in which splicing remains broadly functional during infection while specific transcripts undergo altered splice-site usage or intron retention [40,113–116]. ICP27 interacts with SRPK1, recruits it into the nucleus, and alters SR-protein phosphorylation in association with impaired spliceosome assembly [117]. Structural and biochemical experiments demonstrated how ICP27’s RGG region occupies an SRPK1 substrate-binding site and competitively inhibits the kinase [118]. In addition, ICP27 interacts with cellular cleavage and polyadenylation machinery, including CPSF, to inhibit normal 3’-end processing and transcription termination of host genes [119]. Moreover, a recent study showed that HSV ICP27 uses the host splicing factor SRSF3 to inhibit splicing of selected viral transcripts containing specific SRSF3-binding sequences in the exon upstream of the 5’ splice site. SRSF3 also contributes to NXF1-dependent export of these unspliced transcripts. Imaging revealed colocalization of these factors in nuclear speckles and increased retention of NXF1 in nuclear speckles in cells infected with export-defective ICP27 mutants, linking selective RNA processing to the nuclear distribution of the RNA export machinery [120].

HSV-1 infection also alters the abundance and biogenesis of circular RNAs (circRNAs). Most host circRNAs become relatively enriched because vhs-mediated degradation depletes linear mRNAs, rather than because circRNA synthesis increases. In contrast, HSV-1 actively induces circular and novel linear isoforms of NEAT1_2, the long noncoding RNA that scaffolds paraspeckles, while the RNA remains chromatin-associated. Co-expression of the immediate-early proteins ICP22 and ICP27 is sufficient to induce this processing, although depletion of each protein individually did not change these splicing events during infection [121]. These findings raise the possibility that infection-induced processing of NEAT1_2 affects paraspeckle organization, but its functional consequences remain unresolved. HSV-1-derived circRNAs have also been identified. A recent study identified circularization hotspots at several viral loci, including the middle exon of ICP0 and a region within the LAT intron. Their biogenesis is largely resistant to spliceosome perturbation, with host RNA ligases contributing to selected species, indicating that viral RNA circularization can occur through noncanonical pathways distinct from conventional pre-mRNA splicing [122]. Whether the nuclear changes described here also occur during lytic infection of neurons remains outside the scope of this review and requires separate consideration.

## Viral RNA and nuclear export

HSV-1 RNAs are transcribed in the nucleus, and since many viral mRNAs are intronless, they require the cellular export pathway that is more accustomed to spliced mRNAs. The viral protein ICP27 is the main viral factor that binds viral RNA and connects it to host export components. The main mRNA export adaptor NXF1/TAP was shown to directly bind ICP27 and hence is the main export pathway for viral mRNAs [123,124]. The TREX complex protein Aly/REF was also shown to bind ICP27 and contribute to efficient export [123], although viral RNA export continues even in the absence of the protein [125]. Although the main export pathway for HSV-1 RNAs is NXF1-NXT1-dependent, there are indications that a subset of HSV-1 RNAs are probably exported by the Crm1 (Xpo1) export pathway [126]. Crm1 is known as a protein export factor however, it has a large repertoire of cargos, including small nuclear RNAs (snRNAs), small nucleolar RNAs (snoRNAs), ribosomal RNAs (rRNAs), microRNAs, transfer RNAs (tRNAs) and HIV mRNAs. The interaction of HIV mRNAs with Crm1 is indirect and mediated through the HIV protein REV to facilitate the export of unspliced viral mRNAs [127–129]. We have shown that IE viral mRNAs accumulate in nuclear speckles, which act as an intermediate hub before their export from the nucleus [97] (Figure 2 & 3). Blocking mRNA export caused IE transcripts to accumulate in nuclear speckles, and nuclear speckle disassembly strongly impaired export, which supports a structured pathway of the transcripts in the nucleus (Figure 3). Moreover, we have shown the localization of NXF1 in VRCs during infection, where it probably associates with viral mRNAs en route to export.

**Figure 2.**
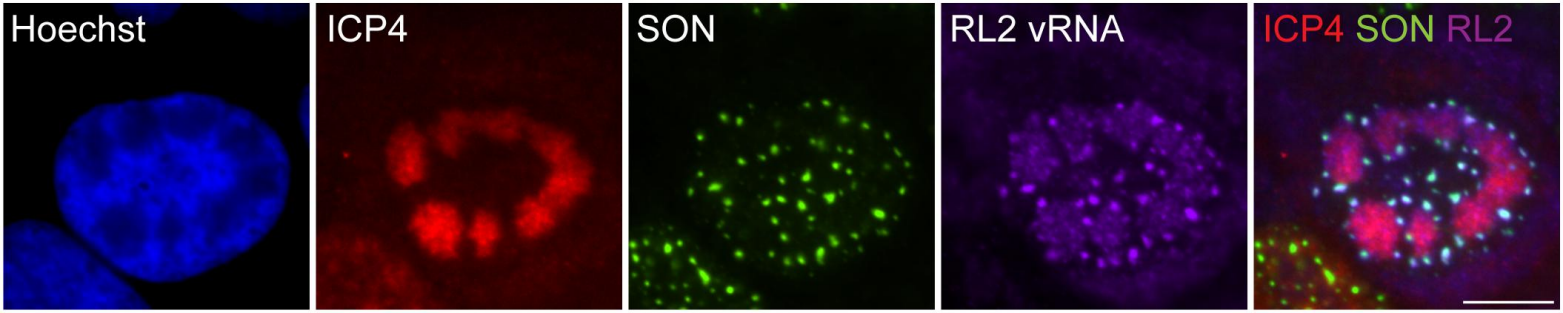
U2OS cell infected with HSV-1 for 6 hours, stained for the viral protein ICP4 (red), which marks the VRCs, and the cellular protein SON (green), which marks nuclear speckles. RL2 viral mRNAs were labeled by RNA FISH (purple). The DNA is counterstained with Hoechst (blue). Scale bar = 10 µm.

**Figure 3.**
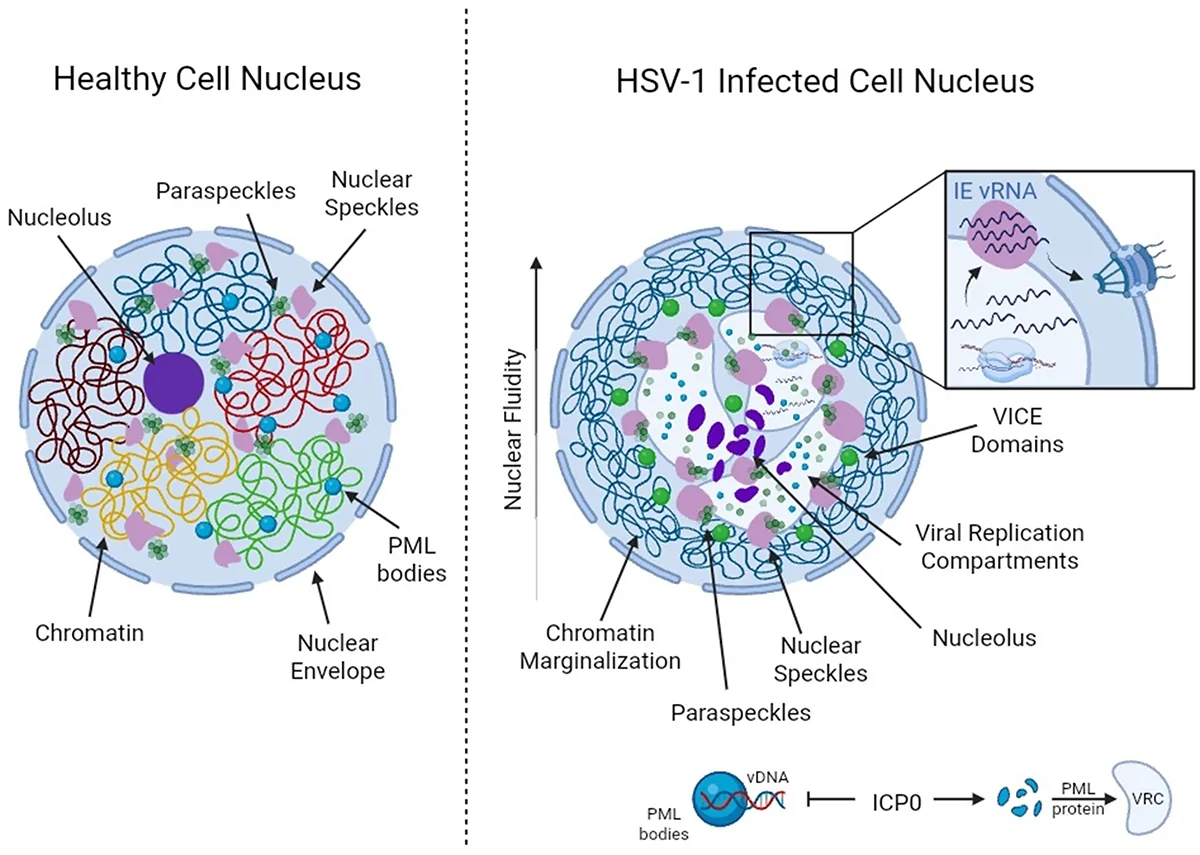
HSV-1–induced remodeling and fluidization of the host cell nucleus. In the uninfected nucleus (left), chromatin is organized within distinct chromosome territories (colored DNA) and the nucleoplasm contains different nuclear bodies e.g. the nucleolus, PML bodies, nuclear speckles and paraspeckles. During productive HSV-1 infection (right), viral replication compartments (VRCs) form and expand, displacing host chromatin toward the nuclear periphery, and redistributing nuclear speckles, which become larger and rounder during infection, around the VRCs. The nucleolus disaggregates, and nucleolar proteins are redistributed, with some recruited to VRCs. Some of the paraspeckle components localize to nuclear speckles whereas some are recruited to VRCs. Virus-induced chaperone-enriched (VICE) domains form adjacent to the VRCs. PML bodies initially capture HSV-1 DNA, but ICP0 inhibits this antiviral restriction, promotes PML body disassembly and PML protein recruitment to VRCs. Immediate-early (IE) viral mRNAs associate with nuclear speckles prior to export through the nuclear pore complex. Collectively, these changes illustrate the extensive reorganization and increased fluidity of the HSV-1-infected nucleus.

In conclusion, HSV-1 mRNA export relies on the host mRNA export machinery, primarily through the direct interaction between the viral protein ICP27 and the cellular export receptor NXF1. However, accumulating evidence suggests that this process is more complex than a simple ICP27-NXF1 coupling mechanism and may also involve additional host factors, including Aly/REF, components of the TREX complex, Crm1-dependent pathways, and nuclear bodies such as nuclear speckles. Notably, immediate-early viral mRNAs, but not early or late transcripts, accumulate in nuclear speckles, raising the question of whether nuclear speckle association reflects a specific requirement of IE transcripts rather than a general feature of HSV-1 mRNA export. Similar to cellular responses to stress conditions, such as heat shock and oxidative stress, in which selected transcripts can still be processed and exported despite widespread suppression of cellular gene expression, HSV-1 late transcripts may exploit selective RNA processing and nuclear export mechanisms to ensure their delivery to the cytoplasm for translation [130]. Future studies should determine whether nuclear speckles facilitate the rapid export or processing of IE mRNAs and how this potential specialization contributes to the ordered progression of viral gene expression.
